# Chest wall perforator flaps are safe and can decrease mastectomy rates in breast cancer surgery: multicentre cohort study

**DOI:** 10.1093/bjs/znae266

**Published:** 2024-11-01

**Authors:** Andreas Karakatsanis, Farid Meybodi, Eirini Pantiora, Elisabeth Elder, Faustine Cabel, Jeremy Hsu, James French, Iliana Aristokleous, Olivia Sjökvist, Daniel Önefäldt, Jaime Navia, Rachel L O’Connell, Jennifer E Rusby, Peter A Barry

**Affiliations:** Department for Surgical Sciences, Uppsala University, Uppsala, Sweden; Section for Breast Surgery, Department of Surgery, Akademiska University Hospital, Uppsala, Sweden; Westmead Breast Cancer Institute, Westmead Hospital, Westmead, New South Wales, Australia; The University of Sydney, Sydney, Australia; Department for Surgical Sciences, Uppsala University, Uppsala, Sweden; Section for Breast Surgery, Department of Surgery, Akademiska University Hospital, Uppsala, Sweden; Westmead Breast Cancer Institute, Westmead Hospital, Westmead, New South Wales, Australia; The University of Sydney, Sydney, Australia; Westmead Breast Cancer Institute, Westmead Hospital, Westmead, New South Wales, Australia; The University of Sydney, Sydney, Australia; Westmead Breast Cancer Institute, Westmead Hospital, Westmead, New South Wales, Australia; The University of Sydney, Sydney, Australia; Westmead Breast Cancer Institute, Westmead Hospital, Westmead, New South Wales, Australia; The University of Sydney, Sydney, Australia; Department for Surgical Sciences, Uppsala University, Uppsala, Sweden; Section for Breast Surgery, Department of Surgery, Akademiska University Hospital, Uppsala, Sweden; Department for Surgical Sciences, Uppsala University, Uppsala, Sweden; Section for Breast Surgery, Department of Surgery, Akademiska University Hospital, Uppsala, Sweden; Department of Plastic Surgery, Akademiska University Hospital, Uppsala, Sweden; Department for Surgical Sciences, Uppsala University, Uppsala, Sweden; Section for Breast Surgery, Department of Surgery, Akademiska University Hospital, Uppsala, Sweden; Department of Plastic Surgery, Akademiska University Hospital, Uppsala, Sweden; Section for Breast Surgery, Department of Surgery, Akademiska University Hospital, Uppsala, Sweden; Breast Unit, Royal Marsden Hospital, London, UK; The Institute of Cancer Research, London, UK; Breast Unit, Royal Marsden Hospital, London, UK; The Institute of Cancer Research, London, UK; Breast Unit, Royal Marsden Hospital, London, UK; The Institute of Cancer Research, London, UK

## Abstract

**Background:**

Chest wall perforator flaps are emerging in oncoplastic breast conservation, mostly as an alternative to mastectomy. However, standardization and consensus on patient selection, techniques, and outcomes have not yet been reached. The aim of this international multicentre collaborative study was to explore practice patterns and outcomes in high-volume centres from different countries.

**Methods:**

Patients with both pre-invasive and invasive breast cancer treated at the Uppsala University Hospital in Uppsala, Sweden, the Royal Marsden Hospital in London, UK, and the Westmead Breast Cancer Institute in Sydney, Australia, were included in this study. The rationale for offering chest wall perforator flaps and surgical outcomes were prospectively documented.

**Results:**

In total, 603 patients were analysed median age of 54 (interquartile range (i.q.r.) 48–63) years, median BMI of 25.0 (i.q.r. 22.5–28.1) kg/m^2^, median tumour extent of 30 (IQR 19–45) mm, median breast volume of 280 (i.q.r. 216–430) ml, and median calculated resection ratio of 16% (i.q.r. 9%–28%). In 67.7%, the treating surgeon had offered chest wall perforator flaps to avoid mastectomy. The procedure was performed as day surgery in 69.5% of patients, with an overall complication rate of 8.6% and the majority of complications being classified as Clavien–Dindo grade I (5.3% of patients). The re-excision rate was 15.9%, with only 1.5% of patients converting to a mastectomy. There were no flap losses. At a median follow-up of 22 (range 12 to 98) months, rates of local recurrence, distant recurrence, and breast cancer-related mortality were 1.9%, 4.9%, and 1.7% respectively.

**Conclusion:**

Chest wall perforator flaps are a useful option to allow more women to avoid mastectomy. In experienced hands, the procedure is safe and should be offered to suitable patients.

## Teaser

In this multinational collaborative study, comprising 603 women with breast cancer from Sweden, the UK, and Australia, it is shown that chest wall perforator flaps are effective with regards to avoiding mastectomy in women without any other technical alternatives. The procedure was undertaken as day surgery in 69% of patients, with a perioperative morbidity of 3.3%. Conversion to mastectomy was as low as 1.5% and short-term follow-up was reassuring in terms of local control, risk of distant metastasis, and breast cancer-specific survival.

## Introduction

Breast-conserving surgery (BCS) followed by radiotherapy has become the standard of care for most patients with pre-invasive and invasive breast cancer. Landmark RCTs have established equivalent survival to mastectomy, whereas recent observational data even suggest a survival advantage^1–3^. Furthermore, BCS has been shown to be superior to mastectomy in terms of patient satisfaction, function, and health-related quality of life^4–6^.

**Fig. 1:**
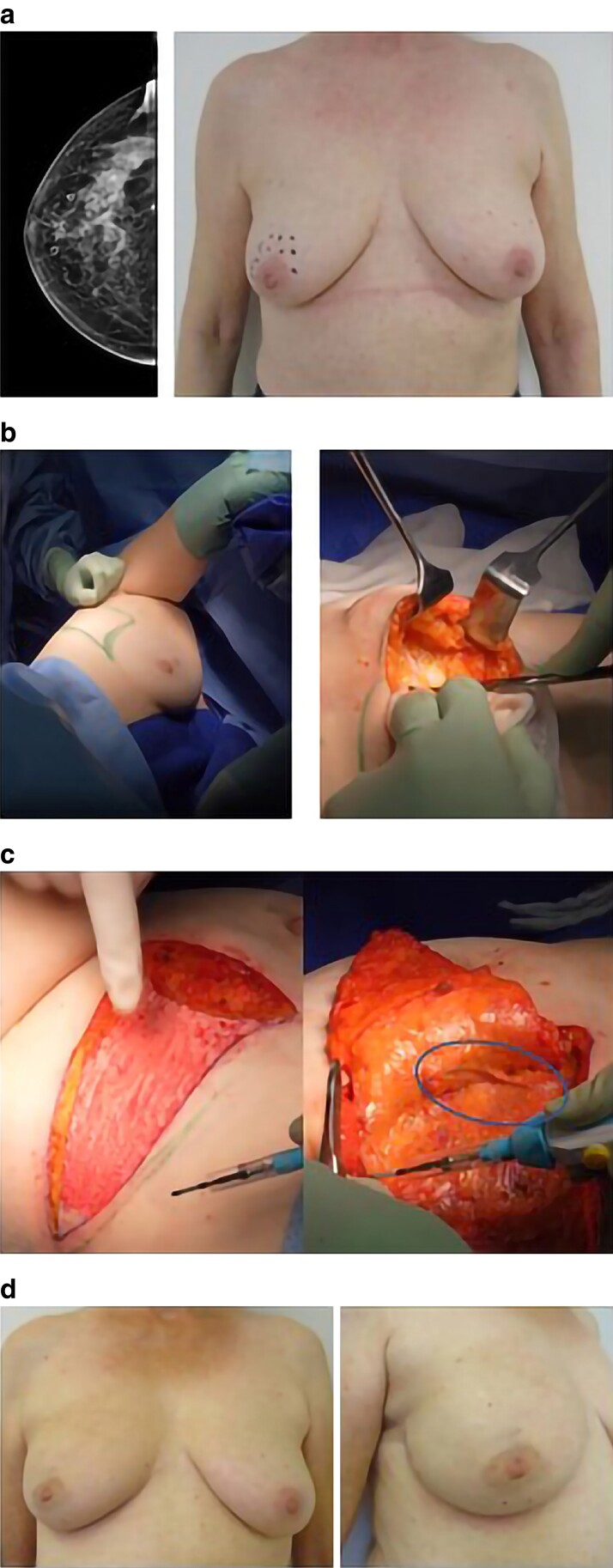
An example of chest wall perforator flap reconstruction **a** A 65-year-old patient with left-sided, retro-areolar cT3 (78 mm), cN0, hormone receptor-negative, ERBB2 (previously HER2)-enriched, invasive ductal (no special type) breast cancer. After primary systemic therapy, the tumour had a complete radiological response, but microcalcifications remained in the area of the footprint. The breast was 395 ml (Longo volume), with grade 1 ptosis, and the intended resection area (target area) was 168 ml, corresponding to a calculated resection ratio of 42.5%. **b** After mapping with Doppler ultrasonography, the perforators were identified and a lateral thoracic artery perforator-based flap containing the perforator sites was marked. The patient was placed in a lateral position (left side up) and subcutaneous dissection was performed through a lateral incision until the area of the tumour. **c** After tumour resection and sentinel lymph node biopsy, the defect and the pivot points were estimated. The flap was de-epithelialized and raised from the back forward, leaving the underlying fascia intact. The circulation of the flap was based on two lateral thoracic artery perforator vessels (oval) and the flap was turned over to fill the defect. **d** Outcome 1 year after surgery and adjuvant radiotherapy.

Oncoplastic BCS (OPBCS) facilitates removal of larger lesions, while achieving excellent functional and aesthetic outcomes, expanding conservation possibilities^7–9^. Volume displacement through various therapeutic mammoplasty techniques is widely available. However, this is not the case for volume replacement, wherein partial breast defects are replaced by tissue from outside the breast. Chest wall perforator flaps (CWPF) (*Fig. 1*) are pedicled adipocutaneous flaps based on the lateral thoracic, thoracodorsal perforator, or intercostal vessels lateral to the breast or under the inframammary fold. Their use for partial breast conservation was described some 20 years ago and, despite growing interest and increasing popularization, high-quality data with regards to patient selection, technique, and outcomes are still scarce^10–12^. A recent large-volume international survey demonstrated that 88.4% of respondents considered CWPF a necessary technique, but only one-third offered the procedure^13^.

Identifying the impact of different patterns in patient selection, indications, and technical variations on outcomes is paramount for further standardization of this reconstructive method. This is particularly true for patients who would otherwise require a mastectomy. Previous single-centre and multicentre retrospective studies have suggested that CWPF can reduce mastectomy rates, with good surgical outcomes, even in women with smaller breasts^14,15^. However, the lack of prospective registration of surgical rationale and the absence of standardized and objective reporting of factors that affect surgical decision-making pose a challenge in the adoption of the method as an alternative to mastectomy.

The aim of this international multicentre collaborative study was to assess the outcomes of CWPF as a surrogate for mastectomy in patients with pre-invasive and invasive breast cancer in centres with experience using the technique.

## Methods

This study was a subset of the ‘Oncologic, Cosmetic and Patient Reported Outcomes in Value-Based Breast Surgery’ (OnCoPRO Value) cohort study (NCT06401304). To assess eligibility for participation, the primary investigators of the PERDITA (PERforator flaps: Doctors needs In Training and Attitudes) Consortium (A.K. and P.A.B.) sought other centres with experience in CWPF, and the following prerequisites: prospective institutional data registration/documentation; a multidisciplinary breast cancer service in university and/or teaching hospitals; breast surgeons with oncoplastic training beyond the learning curve or with guidance from a mentoring surgeon; inclusion of consecutive patients, after the completion of the learning curve for each participating surgeon, and no previous publication of any material. Regarding data requirements, apart from patient, tumour, pathology, and treatment data, it was obligatory for the following to be documented: breast volume (radiological/clinical measurements using any of the Fung, Katariya, Kalbhen, or Longo formula)^16,17^; intended excision volume; the reason for which CWPF were offered (to avoid mastectomy, to avoid asymmetry for patients who did not want contralateral symmetrization, or to avoid a wide local excision or a simpler oncoplastic procedure with inadequate margins and/or suboptimal cosmesis). The units that responded and fulfilled the criteria were: Akademiska University Hospital (UAS), Uppsala, Sweden; Royal Marsden Hospital (RMH), London, UK; and Westmead Breast Cancer Institute (WMD), Sydney, Australia.

After data collection, the harmonization procedure included calculation of the optimal resection volume (ORV), defined as the volume that needed to be resected to remove the lesion with 1-cm radiological margins, using the ellipsoid formula, as suggested in previous literature^18^. Subsequently, the calculated resection ratio (CRR) was defined (CRR = ORV/breast volume). This, alongside tumour location, multifocality/multicentricity, histology, surgery upfront or after neoadjuvant systemic treatment, and patient-related factors (BMI, smoking, diabetes mellitus, and Charlson co-morbidity index^19^), were used to explore how surgical decision-making was affected. The clinical T and N stages at diagnosis were registered based on clinical examination and radiological findings. In cases of multiple ipsilateral lesions (multifocal/multicentric), the clinical T stage was defined by the index lesion, but the extent was that of the footprint that would have to be removed. Given that the perception of ‘alternative to mastectomy’ is prone to subjectivity primarily regarding a satisfactory postoperative result, all surgical assessments from each unit were cross-validated by the local primary investigators from another site (A.K., F.M., and P.A.B.). The acceptable margin was that of the respective national guidelines (no ink on tumour for invasive cancer in UAS and WMD and 1 mm in RMH; 2 mm for pure ductal carcinoma *in situ* in all three centres). To mitigate variability, a sensitivity analysis was performed to see whether a 1-mm margin would have an impact on re-excision frequency.

### Sample size calculation and outcomes

The hypothesis was that CWPF reconstruction is a non-inferior alternative to mastectomy. As ‘avoidance’ corresponds to 100% reduction, a 5% non-inferiority margin was accepted (that is no more than 5% would finally need a mastectomy). The one-sided *P* value was set to 0.025. The type II error (false negative) was set to 0.05, corresponding to 95% power. Consequently, after cross-validation of indications, the ‘need for mastectomy’ cohort would require a minimum of 299 patients. To define ‘need for mastectomy’ more objectively, factors associated with this were sought after cross-validation, through logistic regression and by constructing a nomogram. The ‘need for mastectomy’ cohort, in which the primary endpoint was tested, included only patients that had at least 75% probability of belonging there as per surgeon assessment (*Supplementary Methods*).

The primary outcome was conversion to mastectomy. Secondary outcomes were: re-excision with breast conservation as the final result; practice patterns (procedure as day surgery; use of antibiotics; use of drains); surgical complications per the Clavien–Dindo classification and the comprehensive complication index^20,21^; and time-to-event outcomes, specifically local recurrence-free survival (defined as survival without any isolated ipsilateral breast cancer recurrence), disease-free survival (defined as survival without any distal relapse, regardless of synchronous locoregional relapse), breast cancer-specific survival (defined as time from surgery to death due to breast cancer), and overall survival.

Patient characteristics are presented as median (interquartile range (i.q.r.)) for continuous variables and *n* (%) for categorical variables unless otherwise indicated. Univariable analyses were performed for measures of association and multivariable analyses were subsequently performed for significant associations, with effect sizes presented as OR (95% c.i.). Time-to-event outcomes are presented as HR (95% c.i.), after log rank Kaplan–Meier and Cox regression analysis, and values were adjusted for Charlson co-morbidity index, cT and pT stages, multifocality, cN and pN stages, hormone receptor status, nuclear grade, and receipt of systemic therapy (endocrine therapy, chemotherapy, and human epidermal growth factor receptor 2-targeted therapy). For the exploratory analysis of time-to-event outcomes, patients with a follow-up of less than 12 months were excluded. Analyses were performed using SPSS^®^ (IBM, Armonk, NY, USA) version 28 and Stata version 17. The manuscript was prepared according to the STROBE statement (the STROBE checklist is included in the *Supplementary material*)^22^.

## Results

The total cohort comprised 603 women (median age of 54 (i.q.r. 48–63) years and median BMI of 25.0 (i.q.r. 22.5–28.1) kg/m^2^). The median extent was 30 (i.q.r. 19–45) mm, ranging from 10 to 125 mm, 155 patients (25.7%) had multifocal lesions, the median breast volume was 280 (i.q.r. 216–430) ml, and the median CRR was 16% (i.q.r. 9%–28%) (*Table 1*).

**Table 1:** Patient characteristics

	Total	UAS	RMH	WMD	*P*
Age (years), median (i.q.r.)	54 (48–63)	52 (46–60)	54 (48–62)	59 (50–66)	<0.001*
BMI (kg/m^2^), median (i.q.r.)	25.0 (22.5–28.1)	23.7 (21.5–25.6)	25.0 (22.9–29.1)	26.8 (24.2–30.7)	<0.001*
Charlson co-morbidity index, median (i.q.r.)	3 (3–4)	3 (2–3)	3 (3–5)	4 (3–4)	<0.001†
**Laterality**					
Right	293 (48.6)	119 (44.0)	56 (47.9)	127 (53.8)	0.095‡
Left	310 (51.4)	140 (56.0)	61 (52.1)	109 (46.2)	
Target area extent (mm), median (i.q.r.)	30 (19–45)	30 (19–46)	45 (32–61)	24 (16–32)	<0.001*
**Location in the breast**					
UOQ	339 (56.2)	135 (54.0)	31 (26.5)	173 (73.3)	<0.001‡
Junction UOQ–LOQ	21 (3.5)	10 (4.0)	11 (9.4)	0 (0.0)	
LOQ	90 (14.9)	41 (16.4)	10 (8.5)	39 (16.5)	
Junction LOQ–LIQ	19 (3.2)	2 (0.8)	17 (14.5)	0 (0.0)	
LIQ	40 (6.6)	20 (8.0)	11 (9.4)	9 (3.8)	
Junction LIQ–UIQ	9 (1.5)	3 (1.2)	6 (5.1)	0 (0.0)	
UIQ	20 (3.3)	9 (3.6)	1 (0.9)	10 (4.2)	
Junction UIQ–UOQ	10 (1.7)	5 (2.0)	5 (4.3)	0 (0.0)	
Retro-areolar	13 (2.2)	2 (0.8)	10 (8.5)	1 (0.4)	
Multifocal/multicentric (two or more quadrants occupied)	42 (7.0)	23 (9.2)	15 (12.8)	4 (1.7)	
Breast volume (ml), median (i.q.r.)	280 (216–430)	241 (188–324)	280 (160–370)	415 (280–440)	<0.001*
Optimal resection volume (ml), median (i.q.r.)	45 (28–80)	54 (30–92)	64 (33–113)	38 (24–53)	<0.001*
Calculated resection ratio (%), median (i.q.r.)	16 (9–28)	23 (13–38)	25 (14–47)	11 (6–16)	<0.001*
**Smoking**					
No	553 (91.9)	227 (91.2)	103 (88.0)	223 (94.5)	0.096‡
Yes	44 (8.1)	22 (8.8)	14 (12.0)	13 (5.5)	
**Axillary status**					
Node negative	480 (79.6)	198 (79.2)	104 (88.9)	178 (75.4)	0.009‡
Node positive	123 (20.4)	52 (20.8)	13 (11.1)	58 (24.6)	
**Clinical T stage at presentation**					
T0	58 (9.6)	40 (16.0)	4 (3.4)	14 (5.9)	<0.001‡
T1	265 (43.9)	87 (34.8)	77 (65.8)	101 (42.8)	
T2	237 (43.9)	96 (38.4)	33 (28.2)	108 (45.8)	
T3	43 (7.1)	27 (10.8)	3 (2.6)	13 (5.5)	
**Histology**					
IDC (NST)	411 (68.2)	144 (57.6)	91 (77.8)	176 (74.6)	<0.001‡
ILC	63 (10.4)	24 (9.6)	14 (12.0)	25 (10.6)	
DCIS or pLCIS	58 (9.6)	40 (16.0)	4 (3.4)	14 (5.9)	
Other invasive (tubular, mucinous, metaplastic, etc.)	62 (10.3)	41 (16.4)	2 (1.7)	19 (8.1)	
Mixed IDC/ILC	11 (1.8)	1 (0.4)	8 (6.8)	2 (0.8)	
**Multifocality**					
Yes	155 (25.7)	50 (20.0)	82 (70.1)	23 (9.7)	<0.001‡
No	448 (74.3)	200 (80.0)	35 (29.9)	213 (90.3)	
**Nuclear grade**					
1	86 (14.3)	30 (12.1)	17 (14.5)	39 (16.5)	0.002‡
2	320 (53.2)	129 (52.0)	79 (67.5)	112 (47.5)	
3	125 (32.4)	89 (35.9)	21 (17.9)	85 (36.0)	
**Hormone receptor status**					
Negative	92 (16.3)	37 (17.5)	6 (5.1)	49 (20.8)	<0.001‡
Positive	473 (83.7)	175 (82.5)	111 (94.9)	187 (79.2)	
**erbb2 (previously HER2) status**					
Normal	479 (84.8)	181 (85.4)	97 (82.9)	201 (85.2)	0.825‡
Overexpressed	86 (15.2)	31 (14.6)	20 (17.1)	35 (14.8)	
**Treatment strategy**					
Neoadjuvant systemic therapy	128 (21.2)	58 (23.2)	14 (12.0)	56 (23.7)	0.024‡
Primary surgery	475 (78.8)	192 (76.8)	103 (88.0)	180 (76.3)	

Values are *n* (%) unless otherwise indicated. *Kruskal–Wallis test. †Independent samples median test. ‡Pearson’s chi-squared test. UAS, Akademiska University Hospital (Uppsala, Sweden); RMH, Royal Marsden Hospital (London, UK); WMD, Westmead Breast Cancer Institute (Sydney, Australia); i.q.r., interquartile range; UOQ, upper outer quadrant; LOQ, lower outer quadrant; LIQ, lower inner quadrant; UIQ, upper inner quadrant; IDC, invasive ductal carcinoma; NST, no special type; ILC, invasive lobular carcinoma; DCIS, ductal carcinoma *in situ*; pLCIS, pleomorphic lobular carcinoma *in situ*; IDC, invasive ductal carcinoma; ILC, invasive lobular carcinoma; HER2, human epidermal growth factor receptor 2.

Avoiding mastectomy was the most common indication to offer CWPF reconstruction (408 of 603 patients (67.7% (95% c.i. 63.8% to 71.4%))). The associated factors were BMI, target area to be removed, breast volume, and CRR (*Table 2*). Subsequently, the ‘need for mastectomy’ cohort according to the nomogram (*Fig. S1*; that is patients with greater than or equal to 75% probability of belonging in this group) comprised 301 patients (49.9% (95% c.i. 45.6% to 54.0%)) and differed significantly from the initial assessment (difference 17.9% (95% c.i. 14.2% to 17.7%); *P* < 0.001). In the ‘need for mastectomy’ cohort, key patient characteristics differed significantly (*Table S1*). Specifically, the breast volume was smaller (251 *versus* 326 ml), the target area was larger (41 *versus* 25 mm), the CRR was larger (28.2% *versus* 12.2%), and there were more adverse tumour locations and multifocal/multicentric tumours.

**Table 2:** Characteristics of patients in ‘need for mastectomy’ as per surgeon assessment

	Need for mastectomy				
	Univariable analysis			Logistic regression	
	Yes	No	*P*	OR (95% c.i.)	*P*
Age (years), median (i.q.r.)	52 (46–60)	58 (50–66)	<0.001*	0.999 (0.979, 1.021)	0.961
BMI (kg/m^2^), median (i.q.r.)	24.4 (22.0–27.3)	25.9 (23.1–29.1)	<0.001*	1.080 (1.023, 1.141)	0.006
**Location**					
UOQ	126 (37.2)	213 (62.8)	<0.001†	Reference category	–
Junction UOQ–LOQ	19 (90.5)	2 (9.5)		7.580 (0.822, 69.896)	0.074
LOQ	41 (45.6)	49 (54.4)		1.493 (0.810, 2.752)	0.199
6 o’clock	17 (89.5)	2 (10.5)		2.457 (0.429, 14.082)	0.313
LIQ	26 (65.0)	14 (35.0)		2.634 (0.949, 7.306)	0.063
Junction LIQ–UIQ	8 (88.9)	1 (11.1)		3.096 (0.318, 30.168)	0.331
UIQ	8 (40.0)	12 (60.0)		1.455 (0.413, 5.126)	0.560
12 o’clock	10 (100.0)	0. (0.0)		2.316 (0.267, 20.069)	0.446
Retro-areolar	11 (84.6)	2 (15.4)		1.997 (0.266, 2.361)	0.443
Multifocal/multicentric (two or more quadrants occupied)	34 (81.0)	8 (19.0)		0.792 (0.266, 2.361)	0.676
**Treatment sequence**					
Neoadjuvant systemic therapy	63 (48.1)	68 (51.9)	0.694†	Reference category	–
Primary surgery	236 (50.1)	235 (49.9)		1.071 (0.605, 1.896)	0.813
**Histology**					
IDC (NST)	194 (48.3)	208 (51.7)	0.288†	0.679 (0.331, 1.393)	0.291
ILC	28 (44.4)	35 (55.6)		1.147 (0.486, 2.706)	0.754
Other invasive	40 (54.8)	33 (45.2)		0.095 (0.013, 0.691)	0.020
DCIS or pLCIS	38 (58.5)	27 (41.5)		0.550 (0.270, 1.127)	0.103
Largest extent (mm), median (i.q.r.)	40 (27–55)	22 (15–31)	<0.001*	1.038 (1.022, 1.142)	0.006
Breast volume (ml), median (i.q.r.)	243 (166–280)	430 (280–440)	<0.001*	0.990 (0.988, 0.993)	<0.001
CRR (%), median (i.q.r.)	27.2 (19.4–45.9)	9.7 (6.3–13.9)	<0.001*	142.4 (4.8, 4281.9)	0.004
**Study site**					
UAS	196 (78.4)	54 (21.6)	<0.001†	Reference category	–
RMH	103 (88.0)	14 (12.0)		1.017 (0.396, 2.607)	0.973
WMD	109 (46.2)	127 (53.8)		0.967 (0.552, 1.694)	0.907

Values are *n* (%) unless otherwise indicated. *Mann–Whitney *U* test. †Pearson’s chi-squared test. i.q.r., interquartile range; UOQ, upper outer quadrant; LOQ, lower outer quadrant; LIQ, lower inner quadrant; UIQ, upper inner quadrant; IDC, invasive ductal carcinoma; NST, no special type; ILC, invasive lobular carcinoma; DCIS, ductal carcinoma *in situ*; pLCIS, pleomorphic lobular carcinoma *in situ*; CRR, calculated resection ratio (corresponding to optimal resection volume divided by breast volume); UAS, Akademiska University Hospital (Uppsala, Sweden); RMH, Royal Marsden Hospital (London, UK); WMD, Westmead Breast Cancer Institute (Sydney, Australia).

Practice patterns, complications, and re-excision rates are summarized in *Table 3*. The procedure was performed as day surgery in 419 of 603 patients (69.5% (95% c.i. 65.6% to 73.1%)). Intraoperative antibiotics were administered in 522 of 603 patients (86.4% (95% c.i. 83.6% to 89.2%)) and no drains were placed in 411 of 603 patients (68.4% (95% c.i. 64.3% to 71.9%)). In total, 52 of 603 patients had a complication (8.6% (95% c.i. 6.5% to 11.1%)), with the majority of complications being classified as Clavien–Dindo grade I (32 of 603 patients; 5.3% (95% c.i. 3.7% to 7.4%)). Factors associated with complications in logistic regression were axillary clearance (OR 4.697 (95% c.i. 1.496 to 14.747); *P* = 0.008), operating time (OR 1.013 (95% c.i. 1.005 to 1.022); *P* = 0.003), and resection involving two or more quadrants (OR 3.576 (95% c.i. 1.474 to 8.676); *P* = 0.005). Perioperative morbidity, defined as a complication classified as Clavien–Dindo grade greater than or equal to II, occurred in 20 of 603 patients (3.3% (95% c.i. 2.0% to 5.1%)) and was only associated with smoking in regression analysis (OR 5.075 (95% c.i. 1.646 to 15.648); *P* = 0.005); of these 20 patients, 8 returned to theatre because of bleeding (1.3% (95% c.i. 0.6% to 2.6%)).

**Table 3:** Practice patterns, complications, and re-excision outcomes

	Total	UAS	RMH	WMD	*P*
**Indication**					
To avoid mastectomy	408 (67.7)	196 (78.4)	103 (88.0)	109 (46.2)	<0.001*
To avoid volume displacement with asymmetry and need for contralateral surgery	109 (18.1)	35 (14.0)	12 (10.3)	62 (26.3)	
To avoid WLE with poor cosmetic results	86 (14.3)	19 (7.6)	2 (1.7)	65 (27.5)	
**CWPF technique**					
LICAP-based	317 (52.6)	67 (26.8)	33 (28.2)	217 (91.9)	<0.001*
LTAP-based	172 (28.5)	128 (51.2)	44 (37.6)	0 (0.0)	
TDAP-based	25 (4.1)	24 (9.6)	0 (0.0)	1 (0.4)	
AICAP/MICAP-based	89 (14.8)	31 (12.4)	40 (34.2)	18 (7.6)	
**Single vessel-based CWPF**					
No	245 (40.7)	120 (48.0)	81 (69.8)	44 (18.6)	<0.001*
Yes	357 (59.3)	130 (52.0)	35 (30.2)	192 (81.4)	
**Axillary procedure**					
SLND	452 (75.0)	185 (74.0)	99 (84.6)	168 (71.2)	<0.001*
ALND	91 (15.1)	22 (8.8)	14 (12.0)	55 (23.3)	
TAD	20 (3.3)	20 (8.0)	0 (0.0)	0 (0.0)	
None	40 (6.6)	23 (9.2)	4 (3.4)	13 (5.5)	
**Intraoperative antibiotics**					
No	80 (13.3)	79 (31.7)	0 (0.0)	1 (0.4)	<0.001*
Yes	522 (86.7)	170 (68.3)	117 (100.0)	235 (99.6)	
Operating time (min), median (range)	110 (73–136)	117 (102–143)	140 (120–180)	70 (59–98)	<0.001*
**Procedure as day surgery**					
No	296 (49.1)	32 (12.8)	28 (23.9)	236 (100.0)	<0.001*
Yes	307 (50.9)	218 (87.2)	89 (76.1)	0 (0.0)	
**Drainless procedure**					
No	190 (31.6)	22 (8.8)	48 (41.7)	120 (50.8)	<0.001*
Yes	411 (68.4)	228 (91.2)	67 (58.3)	116 (49.2)	
Length of stay (days), median (range)	0 (0–2)	0 (0–2)	0 (0–9)	1 (0–3)	<0.001†
Any complication					
No	551 (91.4)	219 (87.6)	107 (91.5)	225 (95.3)	0.009*
Yes	52 (8.6)	31 (12.4)	10 (8.5)	11 (4.7)	
**Type of postoperative complication**					
Alleged infection	7 (1.2)	4 (1.6)	2 (1.7)	1 (0.4)	0.005*
Verified infection	9 (1.5)	7 (2.8)	2 (1.7)	0 (0.0)	
Symptomatic seroma	15 (2.5)	10 (4.0)	0 (0.0)	5 (2.1)	
Bleeding	12 (2.0)	4 (1.6)	3 (2.6)	5 (2.1)	
Fat necrosis	3 (0.5)	1 (0.4)	2 (1.7)	0 (0.0)	
Wound dehiscence/delayed healing	6 (1.0)	5 (2.0)	1 (0.2)	0 (0.0)	
**Clavien–Dindo grade of complication**					
I	32 (5.3)	22 (8.8)	4 (3.4)	6 (2.5)	0.004*
II	10 (1.7)	7 (2.8)	2 (1.7)	1 (0.4)	
III	10 (1.7)	2 (0.8)	4 (3.4)	4 (1.7)	
**Perioperative morbidity (Clavien–Dindo grade of complication ≥II)**					
No	583 (96.7)	241 (96.4)	111 (94.9)	231 (97.9)	0.314*
Yes	20 (3.3)	9 (3.6)	6 (5.1)	5 (2.1)	
Comprehensive complication index, median (range)	0 (0–33.7)	0 (0–33.7)	0 (0–33.7)	0 (0–33.7)	0.012†
**Any re-excision for margins**					
Yes	96 (15.9)	24 (9.6)	22 (18.8)	50 (21.2)	0.001*
No	507 (84.1)	226 (90.4)	95 (81.2)	186 (78.8)	
**Completion mastectomy**					
Yes	9 (1.5)	1 (0.4)	0 (0.0)	8 (3.4)	0.012*
No	594 (98.5)	249 (99.6)	117 (100.0)	228 (96.6)	

Values are *n* (%) unless otherwise indicated. *Pearson’s chi-squared test. †Kruskal–Wallis test. UAS, Akademiska University Hospital (Uppsala, Sweden); RMH, Royal Marsden Hospital (London, UK); WMD, Westmead Breast Cancer Institute (Sydney, Australia); WLE, wide local excision; CWPF, chest wall perforator flap; LICAP, lateral intercostal artery perforator; LTAP, lateral thoracic artery perforator; TDAP, thoracodorsal artery perforator; AICAP, anterior intercostal artery perforator; MICAP, medial intercostal artery perforator; SLND, sentinel lymph node dissection; ALND, axillary lymph node dissection; TAD; targeted axillary dissection.

Re-excision to achieve adequate margins was performed in 96 of 603 patients (15.9% (95% c.i. 13.1% to 19.1%)). Associated factors according to univariable analysis were study site (UAS 9.6%, RMH 18.8%, and WMD 21.2%; *P* < 0.001), upfront surgery *versus* surgery after neoadjuvant systemic therapy (17.9% *versus* 8.6%; difference 9.3% (95% c.i. 3.3% to 15.3%); *P* = 0.011), and BMI (Spearman’s rho 0.082 (95% c.i. −0.001 to 0.163); *P* = 0.047), but not belonging to the ‘need for mastectomy’ cohort (16.3% *versus* 16.1%; difference 0.2% (95% c.i. −6.2% to 5.8%); *P* = 0.949). Study site retained significance in logistic regression (UAS: reference category; RMH: OR 1.790 (95% c.i. 0.932 to 3.437) (*P* = 0.080); and WMD: OR 2.635 (95% c.i. 1.415 to 4.906) (*P* = 0.002)), as did upfront surgery (OR 2.299 (95% c.i. 1.174 to 4.504); *P* = 0.015), but the two were highly collinear. Histology and multifocality were not associated. There were no cases of invasive cancer with negative resection margins that were less than 1 mm and thus results did not change based on this.

Conversion to mastectomy was performed in 9 of 603 patients (1.5% (95% c.i. 0.7% to 2.8%)) and was only associated with study site (UAS 1 event, RMH 0 events, and WMD 8 events; *P* = 0.012). All conversions were in patients in the upfront setting, but the events were too few to reach significance (1.9% *versus* 0%; difference 1.9% (95% c.i. −0.7% to 3.1%); *P* = 0.117). There was no difference between the ‘need for mastectomy’ cohort and the rest of the patients (3 of 301 patients (1.0%) *versus* 6 of 286 patients (2.1%); difference 1.1% (95% c.i. −0.9% to 3.1%); *P* = 0.278). There was a correlation between completion mastectomy and BMI according to univariable analysis (Spearman’s rho 0.086 (95% c.i. 0.003 to 0.168); *P* = 0.036), that was the only factor to persist on logistic regression (OR 1.126 (95% c.i. 1.011 to 1.254); *P* = 0.031). In the ‘need for mastectomy’ cohort, there were three events (1.0% (95% c.i. 0.2% to 2.8%)) without any associations. This was lower than the pre-specified 5% cut-off (z-statistic 3.192; *P* = 0.0014), meaning that the primary endpoint was satisfied.

The median postoperative follow-up was 22 (i.q.r. 16–39; range 3–98) months. All patients received adjuvant whole breast radiotherapy that was tolerated well, without any flap failures/losses. A total of 22 patients (3.6% (95% c.i. 2.3% to 5.5%)) required revision surgery (13 for a dog ear revision, 7 for lipofilling, and 1 for both; in addition, 1 patient experienced a fall, with concomitant breast and chest wall injury, resulting in a lump, where investigation showed partial fat necrosis in the flap, which was subsequently excised). All revisions were performed within 18 months of index surgery.

For the oncological follow-up, 529 patients had a follow-up greater than or equal to 12 months and were included in the analysis. The median follow-up was 22 (95% c.i. 21 to 23; range 12–98) months. In total, 10 patients presented with a local recurrence (1.9% (95% c.i. 0.9% to 3.4%)), 26 patients presented with a distant recurrence (4.9% (95% c.i. 3.2% to 7.1%)), and breast cancer-related deaths were documented for 8 patients (1.5% (95% c.i. 0.6% to 2.9%)). The single competing event was a patient who developed angiosarcoma with invasion to the chest wall 11 months after neoadjuvant chemotherapy and surgery for a cT3 N0-to-ypT2 N1 triple-negative breast carcinoma.

Time-to-event outcomes are summarized in *Table 4*. The local recurrence risk was higher after surgery for *in situ* carcinoma, hormone receptor-positive disease, and no adjuvant endocrine therapy. Distant recurrence was associated with a higher Charlson co-morbidity index and advanced nodal disease (pN3 stage), whereas no distant recurrences were observed in patients with pure *in situ* carcinoma. A total of eight patients with invasive breast cancer died during follow-up, all hormone receptor-positive, with nodal stage affecting breast cancer-specific survival.

**Table 4:** Time-to-event outcomes

	Cumulative incidence (%)	Unadjusted analysis		Adjusted analysis	
		HR (95% c.i.)	*P*	HR (95% c.i.)	*P*
**Local recurrence**					
Type					
Invasive breast cancer	1.9	Reference category	–	Reference category	–
*In situ* carcinoma	6.1	3.888 (1.00, 15.07)	0.049	14.95 (0.90, 249.25)	0.060
Hormone receptor status					
Negative	1.1	Reference category	–	Reference category	–
Positive	1.8	1.695 (0.21, 13.55)	0.619	52.63 (4.07, 594.60)	0.005
Endocrine therapy					
Yes	1.2	Reference category	–	Reference category	–
No	4.0	3.166 (0.92, 10.96)	0.069	10.20 (1.25, 83.17)	0.030
**Distant recurrence**					
Charlson co-morbidity index	No value per category provided	1.405 (1.10, 1.80)	0.007	1.544 (1.16, 2.05)	0.003
pN stage					
pN0	0.4	Reference category	–	Reference category	–
pN1	0.4	1.01 (0.37, 2.77)	0.978	0.72 (0.25, 2.14)	0.559
pN2	0.9	2.41 (0.70, 8.28)	0.163	1.74 (0.45, 6.80)	0.426
pN3	33.3	9.697 (1.27, 74.19)	0.029	4.54 (0.52, 39.52)	0.171
**Breast cancer deaths**					
pN stage					
pN0	1.6	Reference category	–	Reference category	–
pN+	1.3	2.41 (1.19, 4.87)	0.014	3.47 (1.10, 10.95)	0.034

Adjusted analysis included Charlson co-morbidity index, clinical and pathological T stages after surgery (cT and (y)pT), multifocality, clinical and pathological N stages after surgery (cN and (y)pN), hormone receptor status, nuclear grade, and receipt of systemic therapy (endocrine therapy, chemotherapy, and human epidermal growth factor receptor 2-targeted therapy).

## Discussion

In this multicentre collaborative study, CWPF are shown to help avoid mastectomy in patients not appropriate for other alternatives. Despite differences in practice patterns among participating centres, the method has low rates of complications and re-excisions, and can be safely performed as day surgery, without drains. Finally, short-term follow-up is reassuring with regards to adequate local control, a finding that is in line with previous publications^23^.

Since their introduction, OPBCS and volume replacement, in particular, have expanded the possibilities of breast conservation, at the cost of more extensive and technically demanding reconstructive surgery^24^. However, this has principally been an intuitive advance, as OPBCS does facilitate the removal of larger specimens, while maintaining good functional and aesthetic outcomes, although the literature suggests low evidence certainty regarding these outcomes^25^. The approach used in this study to allow for tailored decision-making does not stem from the previously described ‘levels’ of OPBCS using the less than 20% and 20–50% cut-offs, popularized by Clough *et al*.^26^. Whilst a large body of literature is based on these classifications and algorithms, there is only a handful of reports on how the assessment was actually performed^27^. There is no doubt that these approaches have served technique description, documentation, and training well, but, moving beyond, it seems that implementing standardized surgical assessment by validated input and output measures constitutes a much more individualized and patient-centred approach within the ethos of modern precision surgical oncology. This approach, as previously shown in other reports, allows for more objective assessments and tailored solutions and may also allow for de-escalation of OPBCS complexity, without compromising patient-reported outcomes^28,29^. As such, the present study, as part of the OnCoPRO Value study, attempts a paradigm shift from surgeons who consider techniques according to the quadrant of the breast affected, or their own familiarity with a particular technique, to the tailored assessment of each patient by meticulously reviewing anatomy, tissue properties, tumour characteristics, and radiological extent. This is a rationalized and evidence-based approach, validated and reproducible, allowing surgeons to approach the technique without the potentially negative nuance of terms such as ‘extreme oncoplasty’^23,30^.

The present study attempted to test the hypothesis that CWPF provide an option to avoid mastectomy using prospective documentation of the alternative options if CWPF were not to be used. This was further refined by harmonization of the outcomes through volumetry of the breast and the ‘target area’, rather than imprecise surrogates such as ‘cup size’ or ‘T stage’. This, along with other patient and breast characteristics, allowed for the construction of a nomogram. This nomogram defined a very selected population in which alternatives to mastectomy other than CWPF were not available in reality, given a median breast volume of 250 ml, a resection ratio of 28%, and unfavourable tumour locations (lower pole, medial breast, or multifocal/multicentric lesions with two or more quadrants involved). This approach aimed to mitigate any variability in the participating surgeons’ perceptions of patients who would ‘otherwise need a mastectomy’. In practice though, surgical judgement will be the main tool that affects decision-making, meaning that this should not be overlooked. Therefore, a standardized assessment based on validated tools should be expected to reduce this variability. The fact that CWPF had a similar effect with regards to avoiding mastectomy in the entire cohort and the ‘mastectomy group’, without differences in re-excisions or complications, is reassuring. Differences in re-excision rates among study sites were highly collinear to upfront surgery, meaning that caution is mandated on whether they represent local differences in operating style or decision-making. Moreover, CWPF, especially when implemented as an alternative to mastectomy, should not be expected to yield re-excision comparable to that of an oncocosmetic breast reduction^9^. Furthermore, given the fact that the present study included patients with a poor response to neoadjuvant systemic therapy and large ductal carcinomas *in situ*, the short-term follow-up for local control is reassuring. This suggests that CWPF can be pursued even in cases of large resection ratios, as long as margin control is obtained.

This study has several limitations. Although randomized comparisons are not realistic in the current era of patient engagement and shared decision-making, a control group, preferably patients undergoing volume displacement or mastectomy with immediate reconstruction, would have given additional insights. On the other hand, these groups are clinically distinct, as removal of breast cancer from a large, ptotic breast is often more appropriately achieved by therapeutic mammoplasty. Moreover, this study included patients with challenging characteristics who wished for breast conservation and demonstrates a surgical approach to accommodate this. The alternative of mastectomy with immediate reconstruction in these patients carries higher risks of reconstructive failure, longer inpatient care and leave of absence, and increased financial cost^31–33^. Finally, the oncological follow-up was short, but this was an exploratory analysis and a longer follow-up is intended.

Strengths of this study include the international multicentre collaboration, encompassing diverse populations and different practice patterns, that allowed for an estimation of the effect of these on surgical outcomes of CWPF. Furthermore, the maintenance of prospective databases with implementation of standardized assessment and validated outcome measures ensured that there were no missing data or assumptions that would have been needed, resulting in high-quality, real-world evidence. Finally, although the present study reports outcomes from high-volume centres, participating surgeons had different levels of experience with CWPF. The outcomes of a structured approach however suggest that reasonable generalizability should be expected in proficient hands.

This multinational collaborative study demonstrates that CWPF can help avoid mastectomy in patients without other appropriate alternatives for breast conservation. The procedure can be offered as day surgery, without drains, with acceptable re-excision and complication rates, and reassuring short-term oncological outcomes. The next steps of the PERDITA Consortium project include a Delphi survey to standardize CWPF training, a study focused on patient-reported outcomes comparing CWPF with mastectomy with immediate reconstruction, a technical report, and a multinational collaborative study for prospective registration in an already validated database.

## Supplementary Material

znae266_Supplementary_Data

## Data Availability

Informed consent forms did not give specific written consent for data to be shared for collaborative purposes; data sharing may be possible after new, specific patient consent.
