# Supplementary material for: Chest wall perforator flaps are safe and can decrease mastectomy rates in breast cancer surgery: multicentre cohort study
Source: Br J Surg. 2024 Nov 1;111(11):znae266. doi: 10.1093/bjs/znae266 (PMC11529790; doi:10.1093/bjs/znae266)
Supplement: znae266_Supplementary_Data [file znae266_supplementary_data.docx]

**Chest wall perforator flaps are safe and can decrease mastectomy rates in breast cancer surgery: multicentre cohort study.**

Andreas Karakatsanis^1,2*^, Farid Meybodi^3,4*^, Eirini Pantiora^1,2^, Elisabeth Elder^3,4^, Faustine Cabel^3,4^, Jeremy Hsu^3,4^, James French^3,4^, Iliana Aristokleous^1,2^, Olivia Sjökvist^1,2,5^, Daniel Önefäldt^1,2,5^, Jaime Navia^2^, Rachel Louise O'Connell^6,7^, Jennifer E. Rusby^6,7^, Peter A. Barry^6,7^.

1. Department for Surgical Sciences, Uppsala University, Uppsala, Sweden
2. Section for Breast Surgery, Department of Surgery, Akademiska University Hospital, Uppsala, Sweden
3. Westmead Breast Cancer Institute, Sydney, Australia
4. The University of Sydney, Sydney, Australia
5. Department of Plastic Surgery, Akademiska University Hospital, Uppsala, Sweden.
6. Breast Unit, Royal Marsden Hospital, London, UK

7. The Institute of Cancer Research, London, UK

*Denotes shared first authorship

Corresponding author: Andreas Karakatsanis, Department for Surgical Sciences, Uppsala University, and Section for Breast Surgery, Department of Surgery, Akademiska University Hospital, Uppsala, Sweden. Email: [andreas.karakatsanis@uu.se](mailto:andreas.karakatsanis@uu.se), X-handle: @KarakatsanisA

**Supplementary Materials - Index**

| **Supplementary Methods** |  |
| --- | --- |
| Nomogram Development | *pag. 4* |
|  |  |
| **Supplementary Figures and Tables** |  |
|  |  |
| Supplement: STROBE Checklist | *pag. 4* |
| Supplement Figure 1: Kattan nomogram to identify patients needing mastectomy if chest wall perforator flap reconstruction was not offered | *pag. 5* |
| Supplement Table 1: Patient characteristics “in need of mastectomy” per surgeon assessment and per nomogram outcomes | *pag. 5* |
|  |  |
|  |  |

Supplementary Methods


Nomogram development

To overcome the subjectivity of individualised surgical assessment as to which patients would, if CWPF reconstruction was not available, otherwise need a mastectomy, univariable and subsequent multivariable logistic regression was performed (results shown in Table 2 of the main manuscript). Following regression, we considered the factors that retained significance in logistic regression. Collinearity diagnostics demonstrated that size (largest extent) and calculated resection ratio (CRR) were highly collinear and size was omitted. Subsequently, a Kattan nomogram was generated using the Stata software and the *nomolog* package (Zlotnik A, Abraira V. A general-purpose nomogram generator for predictive logistic regression models. Stata Journal. 2015. Volume 15, Number 2).

Supplement: STROBE Statement—checklist of items that should be included in reports of observational studies

|  | Item No. | Recommendation | Page  No. | Relevant text from manuscript |
| --- | --- | --- | --- | --- |
| **Title and abstract** | 1 | (*a*) Indicate the study’s design with a commonly used term in the title or the abstract | Page 1 | Title |
|  |  | (*b*) Provide in the abstract an informative and balanced summary of what was done and what was found | Page 2 | Abstract |
| Introduction | | | |  |
| Background/rationale | 2 | Explain the scientific background and rationale for the investigation being reported | Page 3 | Introduction |
| Objectives | 3 | State specific objectives, including any prespecified hypotheses | Page 3 | “The present multinational collaborative study aimed to assess the outcomes of CWPF as a surrogate for mastectomy in patients with pre-invasive and invasive breast cancer in centres with experience with the technique.” |
| Methods | | | |  |
| Study design | 4 | Present key elements of study design early in the paper | Page 3 | Methods, First paragraph |
| Setting | 5 | Describe the setting, locations, and relevant dates, including periods of recruitment, exposure, follow-up, and data collection | Page 3 | Methods, First paragraph |
| Participants | 6 | (*a*) *Cohort study*—Give the eligibility criteria, and the sources and methods of selection of participants. Describe methods of follow-up  *Case-control study*—Give the eligibility criteria, and the sources and methods of case ascertainment and control selection. Give the rationale for the choice of cases and controls  *Cross-sectional study*—Give the eligibility criteria, and the sources and methods of selection of participants | Page 3 | Methods, First paragraph |
|  |  | (*b*) *Cohort study*—For matched studies, give matching criteria and number of exposed and unexposed  *Case-control study*—For matched studies, give matching criteria and the number of controls per case | Not relevant | Not relevant |
| Variables | 7 | Clearly define all outcomes, exposures, predictors, potential confounders, and effect modifiers. Give diagnostic criteria, if applicable | Page 3&4 | Methods, Second paragraph |
| Data sources/ measurement | 8* | For each variable of interest, give sources of data and details of methods of assessment (measurement). Describe comparability of assessment methods if there is more than one group | Page 4 | Methods, Second and Third Paragraph |
| Bias | 9 | Describe any efforts to address potential sources of bias | Page 4 | “Given that the perception of … were cross-validated by the local primary investigators (AK, FM, PAB).” |
| Study size | 10 | Explain how the study size was arrived at | Page 4 | “The hypothesis was that… …belonging there per surgeon assessment. “ |

Continued on next page

| Quantitative variables | 11 | Explain how quantitative variables were handled in the analyses. If applicable, describe which groupings were chosen and why | Page 4 | Patient characteristics are summarized by… Stata v17.” |
| --- | --- | --- | --- | --- |
| Statistical methods | 12 | (*a*) Describe all statistical methods, including those used to control for confounding | Page 4 | Patient characteristics are summarized by… Stata v17.” |
|  |  | (*b*) Describe any methods used to examine subgroups and interactions | Page 4 | Patient characteristics are summarized by… Stata v17.” |
|  |  | (*c*) Explain how missing data were addressed |  |  |
|  |  | (*d*) *Cohort study*—If applicable, explain how loss to follow-up was addressed  *Case-control study*—If applicable, explain how matching of cases and controls was addressed  *Cross-sectional study*—If applicable, describe analytical methods taking account of sampling strategy | Page 4 | Patient characteristics are summarized by… Stata v17.” |
|  |  | (*e*) Describe any sensitivity analyses | Page 4 | Patient characteristics are summarized by… Stata v17.” |
| Results | | | | |
| Participants | 13* | (a) Report numbers of individuals at each stage of study—eg numbers potentially eligible, examined for eligibility, confirmed eligible, included in the study, completing follow-up, and analysed | Page 4 | Results, First paragraph |
|  |  | (b) Give reasons for non-participation at each stage |  |  |
|  |  | (c) Consider use of a flow diagram |  |  |
| Descriptive data | 14* | (a) Give characteristics of study participants (eg demographic, clinical, social) and information on exposures and potential confounders | Page 4 | Results, First paragraph |
|  |  | (b) Indicate number of participants with missing data for each variable of interest | Page 3 | “…no missing data…” |
|  |  | (c) *Cohort study*—Summarise follow-up time (eg, average and total amount) | Page 5 | “The median postoperative follow-up was 22 months (iqr 16, 39; range 3-98 months).” |
| Outcome data | 15* | *Cohort study*—Report numbers of outcome events or summary measures over time | Page 4-6 | Results |
|  |  | *Case-control study—*Report numbers in each exposure category, or summary measures of exposure |  |  |
|  |  | *Cross-sectional study—*Report numbers of outcome events or summary measures |  |  |
| Main results | 16 | (*a*) Give unadjusted estimates and, if applicable, confounder-adjusted estimates and their precision (eg, 95% confidence interval). Make clear which confounders were adjusted for and why they were included | Page 4-6 | Results |
|  |  | (*b*) Report category boundaries when continuous variables were categorized | Page 4-6 | Results |
|  |  | (*c*) If relevant, consider translating estimates of relative risk into absolute risk for a meaningful time period | Page 6 | Results |

| Other analyses | 17 | Report other analyses done—eg analyses of subgroups and interactions, and sensitivity analyses | Page 4-6 | Results |
| --- | --- | --- | --- | --- |
| Discussion | | | | |
| Key results | 18 | Summarise key results with reference to study objectives | Page 6 | Discussion: “In this multicentre collaborative cohort study, …with previous publications.” |
| Limitations | 19 | Discuss limitations of the study, taking into account sources of potential bias or imprecision. Discuss both direction and magnitude of any potential bias | Page 7 | Discussion: “ The study has several limitations….is intended.” |
| Interpretation | 20 | Give a cautious overall interpretation of results considering objectives, limitations, multiplicity of analyses, results from similar studies, and other relevant evidence | Page 6&7 | Discussion: “In this multicentre collaborative cohort study, …with previous publications.”  Discussion: “ The approach used in this study….”extreme” oncoplasty.”  Discussion: “ Strengths of the study include… tshould be safe to extrapolate.” |
| Generalisability | 21 | Discuss the generalisability (external validity) of the study results | Page 7 | Discussion: “ Strengths of the study include… tshould be safe to extrapolate.” |
| Other information | |  | | |
| Funding | 22 | Give the source of funding and the role of the funders for the present study and, if applicable, for the original study on which the present article is based | Page 8 | Author Disclosures: “ Funding: No funding was available for this project.” |

Supplemental Figure 1: Kattan nomogram to identify patients needing mastectomy if chest wall perforator flap reconstruction was not offered.


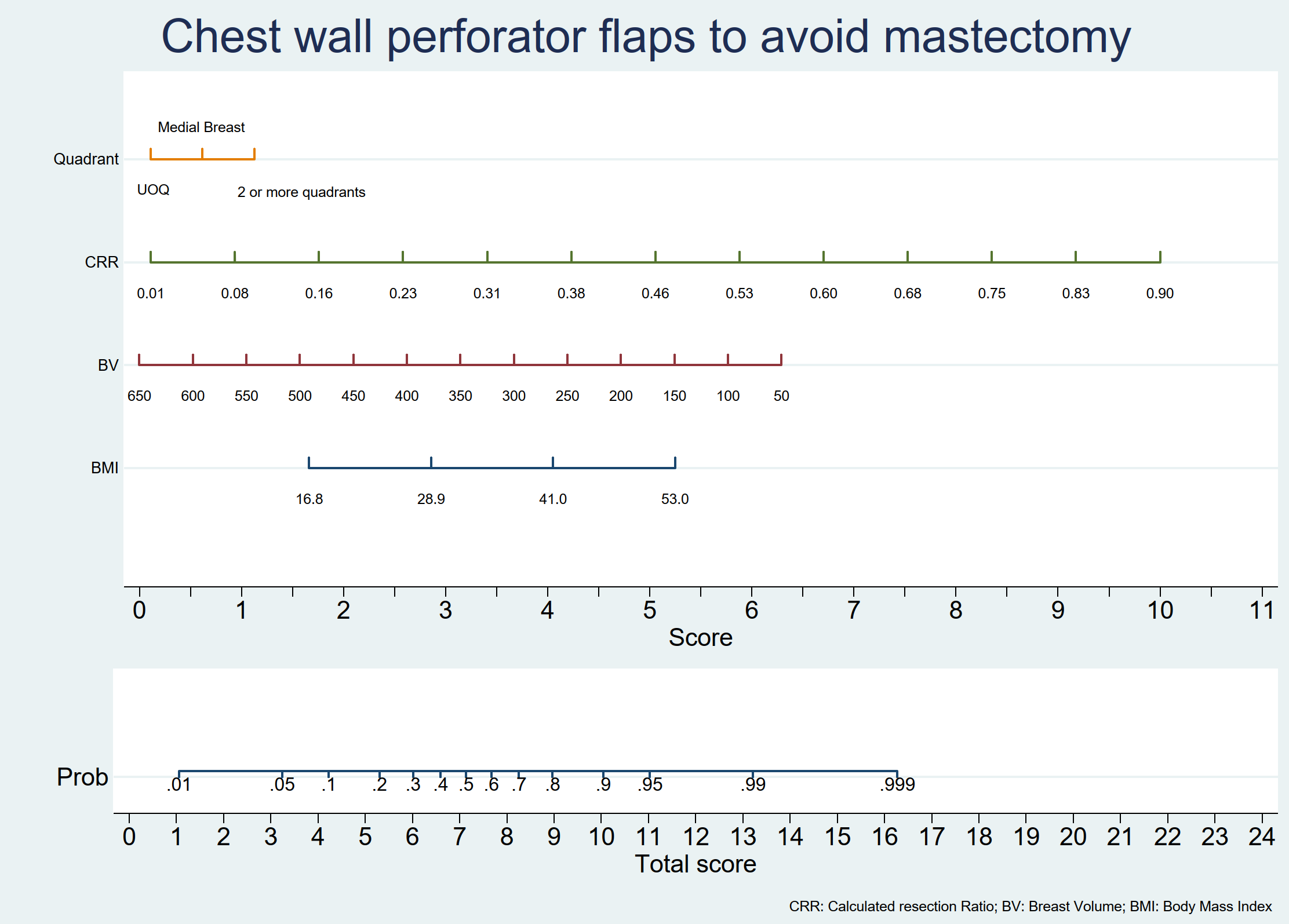


Patients with a score of 8.5 or more, corresponding to a probability of 75% or more of being assessed as “otherwise requiring mastectomy” were assigned to the mastectomy cohort.

Supplement Table 1: Patient characteristics “in need of mastectomy” per surgeon assessment and per nomogram outcomes

|  |  | CWPF to avoid mastectomy |  |  |  |
| --- | --- | --- | --- | --- | --- |
|  |  |  |  |  |  |
|  |  | Clinician assessment (n=408) | Nomogram cut-off <75% (n=107) | Nomogram cut-off >75% (n=301) | p-value |
| Body Mass Index (kg/m2) | | 24.5 (22.1, 27.9) | 24.9 (21.9, 28.7) | 24.4 (22.1, 27.3) | 0.375* |
| Breast Volume (ml) | | 280 (180, 358) | 326 (280, 430) | 251 (160, 280) | <0.001* |
| Target area (mm) | | 35 (23, 50) | 25 (18, 35) | 41 (29, 55) | <0.001* |
| Calculated Resection Ratio (%) | | 21.9 (13.8, 38.3) | 12.2 (8.3, 16.6) | 28.2 (19.4, 46.2) | <0.001* |
| Age (yrs) | | 54 (47, 61) | 56 (47, 68) | 53 (47,60) | 0.004* |
| CCI | | 3 (2,4) | 3 (2,4) | 4 (3,4) | <0.001** |
| Location in the breast | UOQ | 194 ( 100.0) | 80 (41.2) | 114 (58.8) | <0.001*** |
|  | Junction UOQ-LOQ | 19 (100.0) | 1 (5.3) | 18 (94.7) |  |
|  | LOQ | 58 (100.0) | 17 (29.3) | 41 (70.7) |  |
|  | 6 o'clock | 17 (100.0) | 2 (11.8) | 15 (88.2) |  |
|  | LIQ | 31 (100.0) | 5 (16.1) | 26 (83.9) |  |
|  | Junction LIQ-UIQ | 8 (100.0) | 2 (25.0) | 6 (75.0) |  |
|  | UIQ | 11 (100.0) | 3 (27.3) | 8 (72.7) |  |
|  | 12 o'clock | 9 (100.0) | 0 (0.0) | 9 (100.0) |  |
|  | Central / Retroareolar | 11 (100.0) | 2 (18.2) | 9 (81.8) |  |
|  | Multifocal/Multicentric | 37 (100.0) | 4 (10.8) | 33 (89.2) |  |

Continuous and Ordinal Variables are summarised as medians with interquartile range (iqr) and categorical variables as numbers with percentages (%). CCI: Charlson Comorbidity Index, CWPF: Chest Wall Perforator Flap, LIQ: Lower Inner Quadrant, LOQ: Lower Outer Quadrant, ml: millilitres, mm: millimetres, UIQ: Upper Inner Quadrant, UOQ: Upper Outer Quadrant. *: Mann Whitney U-test, **: Independent Samples Median test, ***: Pearson’s Chi-Square test.
